# Role of the membrane receptor ALXR in polymicrobial sepsis

**DOI:** 10.1186/cc11804

**Published:** 2012-11-14

**Authors:** LC Resende Silva, ID Tavares, MM Teixeira, D Da Gloria De Souza

**Affiliations:** 1 ICB/UFMG, Belo Horizonte, Brazil

## Introduction

Sepsis is a leading cause of death in the ICU. It is characterized by a systemic inflammatory response following bacterial infection. Inflammatory dysregulation affects multiple organs through effects on endothelial, epithelial and immune cells, which can lead to irreversible damage and death of the host. Although exacerbated inflammation plays a pivotal role in sepsis evolution, anti-inflammatory drugs fail to treat this disease. In addition, anti-inflammatory therapy may inhibit clearance of infection or favor secondary infections. Therefore, it is of interest to resolve excessive inflammation without compromising host defense mechanisms. In accordance, it has been shown that treatment with LXA4, a endogenous anti-inflammatory agent with pro-resolution activity, increased survival in experimental sepsis by reducing systemic inflammation as well as bacterial spread. We therefore aimed to evaluate the role of a nonspecific LXA4 receptor, ALXR, in host response to sepsis induction by cecal ligation and puncture in mice (CLP).

## Methods

To assess the role of ALXR in the CLP model we used WT (C57BL/6) mice treated or not with an ALXR antagonist, BOC-1 (2 mg/kg, i.v.), 30 minutes before sepsis induction. Then, 6 hours after mild and severe sepsis induction, we evaluated the pulmonary damage, bacterial spread, neutrophil recruitment and the levels of cytokines, chemokines in the lung, peritoneal exudate and plasma.

## Results

Animals submitted to mild and severe sepsis induction and treated with BOC-1 presented decreased neutrophil migration, larger bacterial loads in peritoneal exudate, and heightened lung damage when compared with untreated control animals. Interestingly, ALXR blockade did not alter levels of cytokines and chemokines such as IL-6, IL-1β, TNFα and KC in all evaluated tissues.

## Conclusion

Animals treated with the antagonist of ALXR receptor demonstrated a worse outcome after sepsis induction when compared with animals that received vehicle. These data suggest that the membrane receptor ALXR plays a protective role during response to sepsis induced by CLP.

